# How Do I Look? Body Image Perceptions among University Students from England and Denmark

**DOI:** 10.3390/ijerph7020583

**Published:** 2010-02-21

**Authors:** Walid El Ansari, Agna Rigmor Vodder, Andi Mabhala, Christiane Stock

**Affiliations:** 1 Faculty of Sport, Health and Social Care, University of Gloucestershire, Gloucester, UK; 2 Department of Public Health, University of Copenhagen, Copenhagen, Denmark; 3 Faculty of Health and Social Care, University of Chester, Chester, UK; 4 Unit for Health Promotion Research, Institute of Public Health, University of Southern Denmark, Niels Bohrs Vej 9-10, 6700 Esbjerg, Denmark

**Keywords:** body image perception, student health, quality of life, gender, nutrition

## Abstract

This study examined differences in body image perception between university students in two European countries, United Kingdom and Denmark. A total of 816 British and 548 Danish university students participated in a cross-sectional survey. A self-administered questionnaire assessed socio-demographic information, body image perception (as “too thin”, “just right” or “too fat”), and the association of related factors with body image perception (nutrition behaviour, social support, perceived stressors and quality of life). The proportions of students who perceived themselves as “too thin”, “just right”, or “too fat” were 8.6%, 37.7%, and 53.7% respectively. Multi-factorial logistic regression analysis showed that students who perceived themselves as “too fat” were more likely to be from the British university, to be females, to be older than 30 years, to report stress due to their financial situation and were less likely to have a high quality of life. The findings highlight the need for interventions with focus on healthy food choices whilst acknowledging financial stressors and quality of life.

## 1. Introduction

In the western world, there is an increasing focus on body image. Pictures of movie stars and fashion models strongly impact on girls’ body shape and image perception [1]. Such mass media and diverse socio-cultural pressures are seen to cause an increased awareness of being thin as ideal, and to contribute to the misperception of body weight: how the body is viewed and evaluated by the individual and by others. Hence, the last decades have witnessed surging interest by the academic community in body image [2]. A complex range of factors influences body image perception. These include socio-demographic factors (gender; age; country), nutrition, and psycho-social factors e.g., stress, social support and quality of life.

*Socio-demographic factors (gender, age, country)*: girls are more likely to express weight dissatisfaction than boys, and body weight perception and dissatisfaction are correlates for weight control practices [3]. Indeed, an increasing public health challenge is that 2% to 4% of young adult females have full-syndrome eating disorders that harm their general health and may cause death [4].

Similarly, men too strive to lose weight to conform to today’s ideal body shape. Whilst many studies have investigated body image perception in women [5], less have done so for men [6]. This is despite that men [7] with eating disorders feel considerably more obese than subjects without such conditions. Others have shown wide disagreement between men’s actual muscularity and their body ideals [8], and that some men were alarmed about being overweight, were dissatisfied with their body, and reported an ambition to realize a leaner stature [9]. In relation to age, the association of the age of university students with body image perceptions seem to have not received much attention in the literature, perhaps because of the narrow age bands observed in traditional college student populations.

As for country, satisfaction with and concerns about body weight are affected by social norms and cultural standards [10,11], where being thin is greatly valued within Western societies [12]. Social judgment of appearance seems partly responsible for the unrealistic weight goals sought by young adults [13]. Norms and socio-cultural pressures differ among countries; hence it is likely that the proportions of people dissatisfied with their body image differ between countries [14]. The fact that Denmark belongs to the Scandinavian regime that is characterized by high levels of social protection, comparatively generous social transfers, and state-promoted social equality of the highest standards may have a positive influence on body satisfaction.

*Perceived stressors*: stress has been linked to body weight [15], and is also associated with unhealthy nutrition: stress not only increases food consumption in certain individuals but also shifts their food choices from lower fat to higher fat foods [16]. Thus stress and dissatisfaction with body weight have been reported as key risk factors in the aetiology of eating disorders [17].

*Nutrition behaviours*: nutritional behaviours of university students are similarly critical to body image perceptions. In adolescents, body weight perception is a key determinant of nutritional habits [18], and furthermore, nutritional habits and body-shape preferences vary across cultures [19].

*Social support and satisfaction with social support*: social support plays a vital role in the maintenance of health behaviours and the stimulation of health behaviour modification [20]. Without proper support and coping strategies, people might adopt unhealthy behaviours, such as smoking, alcohol consumption, isolation, irritability, and disruptive eating patterns e.g., [21,22].

This study investigated the factors associated with body image perception among university students in two European countries (United Kingdom and Denmark). One university from each country was included in the study, chosen on the basis of research interests, existing contacts and history of successful previous collaboration. The study aimed to investigate differences in body image perception between students from a British and a Danish university. In addition, we examined the association of socio-demographic factors (gender, age, country) and lifestyle characteristics (perceived stressors, nutrition behaviours, quality of life, social support and satisfaction with social support) with body image perception. We expected more males than females, and more Danish than British students to perceive their bodies as “just right”. We also hypothesized that a low level of perceived stress, a high level of social support, and a higher quality of life would be associated with body image satisfaction.

## 2. Methods

### 2.1. Characteristics of the Study Sample

The sample included 1,414 university students from the University of Chester (UC) in England and from the University of Southern Denmark (SDU) in Denmark. The UC sample (866 students) comprised 76.7% females and 23.3% males, with a mean age of 26.8 years (SD 9.7). The SDU sample (548 students) included 48.7% females and 51.3% males, with a mean age of 23.7 years (SD 6.3). The sample included students from the different faculties and campuses at each of the two universities in order to represent the student distribution. The vast majority of students at both universities had the nationality of the respective country (at UC 96.4% from UK; at SDU 94.0% from Denmark). Response rates to the survey were 89.5% (UC) and 92.3% (SDU).

### 2.2. Data Collection

Data used in the present analysis was collected as part of the Student Health Survey [23] in 2007 at UC and in 2005 at SDU. After ethical approval, self-administered questionnaires were distributed to students during their lectures, and participation was voluntary and anonymous. Classes were selected using convenience sampling method. The selection of classes did include students from all faculties and all campuses at each university. All data were confidential and data protection was observed at all stages of the study.

### 2.3. Questionnaire

The questionnaire included socio-demographic information (gender, age, sex, and financial situation), self-reported health data, as well as questions related to health behaviours, stressors, nutrition, social support and quality of life.

Body image perception was assessed on a five-point Likert scale adapted from the Health Behavior in School-aged Children (HBSC) study [24]. Students were asked: “In your opinion are you…”, with five response options (“Far too thin”, “A little too thin”, “Just right”, “A little overweight”, “Very overweight”). For the analysis, the five options were re-coded into three binary variables (“Too thin”, “Just right”, “Too fat”).

The frequency of perceived stressors was measured with the question “How much have you felt being stressed in the last month by the following factors?” The factors included: studies in general; housing; financial situation; and, workload in addition to studying. These were rated using a 6-point Likert scale in the British questionnaire and a 4-point Likert scale in the Danish questionnaire (from “Never stressed” to “Very often stressed”). For the analysis, a binary variable was created by combining the two or three lower categories to one category “lower stress level” and combining the two or three higher categories to “higher stress level”.

Nutrition behaviour was assessed by a food frequency questionnaire [25] containing the following items: sweets (chocolate, candy, *etc.*)*; cake/cookies*; snacks (chips, peanuts, *etc.*)*; fast food/canned food (pizza, hamburger, French fries, canned ravioli, *etc.*)*; fresh fruit, salad/ raw vegetables; cooked vegetables; and fish/ sea food. Each of these items was measured on a five-point Likert scale: “Several times a day” (1 point), “Daily” (2 points), “Several times a week” (3 points), “1−4 times a month” (4 points) or “Never” (5 points). Using these points all food items marked with * were used to construct a sum score named “High calorie diet score”. The rest of the food items were used to construct a sum score labeled “Healthy diet score” by reversing the point scale (*i.e*., several times a day = 5 points). For the analysis the scores were re-coded into three tertiles: “Low”, “Medium”, and “High” score.

Quality of life was measured by the question: “If you consider the quality of your life: How did things go for you in the last four weeks?” based on the quality of life measurement charts [26] with the 5 response categories ranging from “Very badly” to “Very well”. The variable was further re-coded into three new categories “Low”, “Medium” and “High” quality of life.

Social support was measured by modifying the Sarason’s Social Support Questionnaire [27], using the question: “How many people do you know—including your family and friends—support you whenever you feel down?” The numerical response was re-coded into “Low” (<3 persons) or “High” (≥3 persons) social support. Satisfaction with social support was measured by the question: “Are you on the whole satisfied with the support you get in such situations?” using a 5 point Likert scale, which was re-coded into three categories (“Low”, “Medium” and “High”) for the analysis.

### 2.4. Statistical Analysis

The data was analysed using SPSS statistical package version16.0, with significant level set at *p* < 0.05. Chi-square (χ^2^) test was used to compare the frequencies in the three body perception categories between the two study sites and between males and females. Multi-factorial logistic regression analysis examined the association of the factors gender, age, university, perceived stressors, nutrition behaviour, quality of life, social support and satisfaction with social support with the three body image perceptions as dependent variable (“Too thin”, “Just right”, “Too fat”) using the enter mode and thus controlling for all other factors.

## 3. Results

### 3.1. Characteristics of British and Danish Students

Table 1 shows the main characteristics of the study populations. Compared to the Danish sample, British respondents comprised higher proportions of females and of either young (<20 years) or older (≤30 years) students. Regarding stressors, British students were more likely to perceive the stressors of financial situation and workload in addition to studying compared to Danish respondents. British students scored lower at the “high calorie diet score” and higher at the “healthy diet score” than the Danes. Danish participants reported a higher quality of life than the British counterparts. While there was no difference between the two countries in the quantity of social support, more Danes were satisfied with the support they received.

### 3.2. Perceived Body Image by University and Gender

Figure 1a shows the distribution of perceived body image by gender. More males perceived themselves as “too thin” and “just right”, while females were more likely feel that they were “too fat” (*p* < 0.001). Figure 1b depicts perceived body image by university, where more Danish students perceived their body image as “just right”, whereas more British participants felt “too fat” (*p* < 0.001).

### 3.3. Factors Associated With Body Image Perception

The proportions of students who perceived themselves as “too thin”, “just right” or “too fat” were 8.6%, 37.7%, and 53.7% respectively. Multi-factorial logistic regression analysis examined the associations between socio-demographic and lifestyle factors as independent variables and body image perception (3 categories) as the dependent variable.

The analysis showed that students who perceived themselves as being “too thin” were more likely to be males and less likely to be older than 30 years, having a high calorie diet score or having a high healthy diet score. Students who perceived their body as being “just right” were more likely to be males, to have a high healthy diet score and to have a higher quality of life. In addition, they were less likely to be from the University of Chester and to be stressed by their financial situation. Students who perceived themselves as “too fat” were more likely to be females, to be from the University of Chester to be older than 30 years, to be stressed by their financial situation and less likely to have a high quality of life.

Some factors were not associated with any of the categories of body image perception. These were the perceived stress of studies in general, of the workload in addition to studying and of the housing situation of the participants. Moreover, social support and satisfaction with social support were not associated with body image perception.

## 4. Discussion

This study assessed the factors that are independently associated with body image perception among British and Danish university students, while controlling for all other factors. Below, we only discuss the factors that displayed such significant associations (gender, age, country, perceived stressors, nutrition behaviours, and quality of life).

As regards to gender, the study findings affirmed the expected association between gender and body image perception: males tended to have a more ‘positive’ body image perception compared to females. This is supported by other studies showing that women were more likely to perceive themselves as being overweight than men [28,29]. As our findings suggested, compared to men, women tend to have a more ‘negative’ attitude towards their bodies, and the desire to be thin is a critical factor in women’s outlook toward their bodies and body image perception [30]. Men find a greater variety of body shapes to be socially acceptable than women, whereas women have a narrower range of what is considered the ‘ideal’ body image. Consequently, women more often than men perceive themselves as overweight. Hence, dissatisfaction with one’s weight, and attempting to achieve one’s ideal body shape are seen as risk factors of eating disorders and health-compromising behaviours [31]. However, we found that even though it was mostly women who tended to perceive themselves as “too fat”, more than one third of men also reported feeling “too fat”. This suggested that men too are prone to the perceived ‘problems’ of body dissatisfaction, and hence, as women, might comprise a potential risk-group for the development of eating disorders. It is also noteworthy that almost each fifth man in our sample perceived himself as being “too thin”, a perception that may encourage unfavourable eating practices in the opposite direction such as overeating. Indeed, body image dissatisfaction is of concern for males as well as females, although the distribution is different [32].

In connection with the second socio-demographic factor (age), the only significant associations were for those students aged ≥30 years when compared with those <20 years of age. Across the whole sample, older students were less likely to feel “too thin” and more likely to feel “too fat”. The lack of statistically significant differences could be attributed to that the age difference (span) between the students in our sample was narrow. Within a broader age span, Franzoi [33] found that although men had more positive body images than women in both older and younger age groups, the gender difference becomes less pronounced for those over age of 65. The social attitudes of aging women as unattractive could influence females as regards the actual appearance of their aging bodies in a negative way [34].

Concerning the third socio-demographic factor (university/country), the study findings affirmed the expected association between country and body image perception: more Danish students felt “just right” and more British students felt “too fat”, suggesting higher satisfaction of the Danes with their body image. To the best of our knowledge, no other studies have examined differences in body image perception between strictly British and Danish students, although studies among other countries have been presented. Our findings showed that 20% more British than Danish students felt that they were “too fat”. One potential explanation of this difference might be due to socio-economic and political differences between the two study sites, such as income, gender issues, political models, and social rights, which could act as mediatory factors that moderate attitudes towards thinness and body image ideals. The UK has historically seen a strong masculine breadwinner model, which has portrayed married women primarily as dependent mothers and wives and not as independent workers [35]. Aspects of this norm might still be present and might likely be associated with women’s self-perceived body image. Within the European welfare states, England belongs to the Anglo-Saxon regime where state provision of welfare is minimal and social protection levels are modest. Denmark on the other hand, belongs to the Scandinavian regime that is characterized by high levels of social protection, comparatively generous social transfers, and state-promoted social equality of the highest standards [36]. On the general population level, studies have shown that overall population health tends to be worse in the welfare states of the Anglo-Saxon regime [37–40].

As regards to the first lifestyle characteristic (perceived stressors), students reporting stress due to their financial situation were less likely to feel “just right” and more likely to feel “too fat”. This is in agreement with others [41] who showed a link between daily stress and depressed mood in adolescents and adults. When entering university, financial difficulties can be a contributing factor to stress among students [42]. Due to such expected influences of stress on subjective well-being, it is likely that depressed mood could mediate the effect of financial stress on body image perception, possible causing a negative body image judgement.

In connection with the second lifestyle characteristic (nutrition behaviours), the study revealed that both the “healthy diet score” and the “high calorie diet score” were associated with feeling “too thin”. Further, the “healthy diet score” was associated with students perceiving themselves as “just right”. It is notable that none of the scores were associated with feeling “too fat”. The findings are supported by others confirming that disrupted beliefs about one’s body image can lead to dieting among students. Inappropriate weight concerns and dieting could compromise the quality of food intake [43]. Body image concerns among college students dispose them to food restrictive behaviours and eating disorders [44,45], to the extent that body shape concerns were considered a causal risk factor for eating disorders in college women [4].

With reference to the third lifestyle characteristic (quality of life), the study findings affirmed the association between a higher quality of life and the perception of being “just right”. Moreover, students who perceived themselves as “too fat” reported a lower quality of life. This is supported by findings that better body image was also related to higher self-esteem, optimism and social support among women [34], all of which confirm the importance of quality of life [46]. Quality of life seems to have a positive effect on how students perceive their body image, but the opposite direction of the effect is also likely. Further, this highlights the significance of providing ‘healthy’ settings for students that would be conducive that they feel satisfied with their daily environment.

This study has limitations when considering the generalizability of the findings. Response bias cannot be excluded, as some respondents tend to answer many questions in the same way [47]. Differences between countries could actually be differences between universities. As a cross-sectional survey, the findings are associations not causations, with difficulty in determining the direction of the effects. It would have been beneficial to link students’ perceived body image with their actual Body Mass Index (BMI), but this was not possible, due to lack of data. Therefore it was unfeasible to assess whether reported body image perceptions corresponded with students’ actual body weight or BMI. In addition, some of the measures used, such as the dietary measures and the measure of psychosocial stress were short form measures and had therefore shortcomings. The necessity of a general student health survey to be conducted within short time in classes, however, makes the use of in depth measures for each health factor unfeasible.

## 5. Conclusion

Several important conclusions can be drawn from the results of this study. Factors which were significantly associated with body image perception should be further studied (gender, age, university, nutrition behaviour, financial stressors, quality of life). Interventions among university students should relate actual measured BMI to body image perception of the students in order to target students at risk. Furthermore, interventions should, depending on the relationship between body image perception and actual BMI amongst students, focus on exercise, healthy lifestyle, healthy food choices, altering body image perception, important stressors and quality of life. Universities should offer individual counselling for at risk students in order to prevent eating disorders, and should offer psychological and stress related counselling, but should also counteract unrealistic body image concerns of students by broad health communication campaigns. Moreover, the association between quality of life and body image perception highlights the importance of supporting students throughout their studies, and provide healthy environments, both within the context of university and in their general life.

## Figures and Tables

**Figure 1a.**
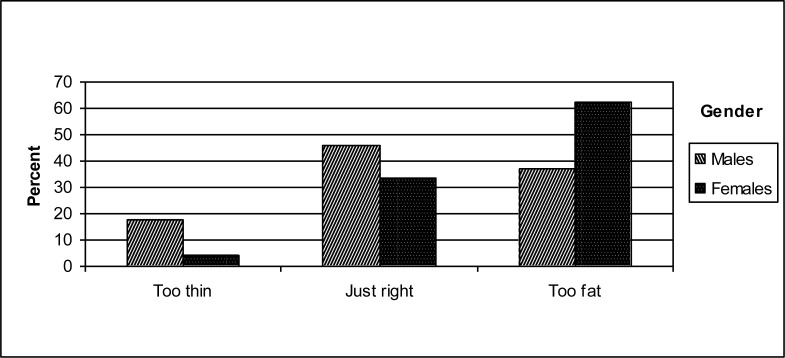
Perceived body image by gender.

**Figure 1b.**
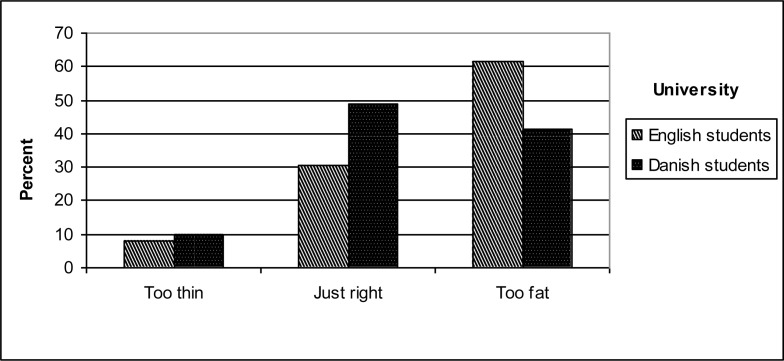
Perceived body image by university.

**Table 1.** Nutrition and lifestyle characteristics of British and Danish students.

**Variable**	**University of Chester** (n = 866)		**University of Southern Denmark** (n = 548)		***p* value** *

	n	%	n	%	
**Gender**					<0.001
Female	626	76.7	267	48.7	
Male	239	23.3	281	51.3	
**Age (year)**					<0.001
<20	241	27.8	29	5.3	
20–24	243	28.1	400	73.0	
25–29	78	9.0	70	12.8	
≥30	304	35.1	49	8.9	
**Perceived stress**					
Studies in general	408	49.0	245	45.5	0.201
Housing	93	11.2	54	9.9	0.463
Financial situation	354	42.8	181	33.2	<0.001
Workload in addition to studying	415	49.2	80	15.0	<0.001
**Nutrition score**					
High calorie diet score^1^					<0.001
Low (1^st^ tertile)	354	45.6	92	17.0	
Medium (2^nd^ tertile)	243	31.3	233	42.9	
High (3^rd^ tertile)	180	23.2	217	40.1	
Healthy diet score^2^					<0.001
Low (1^st^ tertile)	236	30.3	278	51.1	
Medium (2^nd^ tertile)	213	27.3	136	25.0	
High (3^rd^ tertile)	331	42.4	130	23.9	
**Quality of life**					0.007
Low	55	6.5	52	9.9	
Medium	243	28.9	119	22.7	
High	542	64.5	354	67.4	
**Social support**					0.587
Low (<3 persons)	284	33.7	174	32.3	
High (≥3 persons)	559	66.3	365	67.7	
**Satisfaction with social support**					<0.001
Low	75	8.9	31	5.7	
Medium	473	56.4	214	39.6	
High	290	34.6	295	54.6	

* χ^2^-test to compare the two study sites;

1 Low vitamins and minerals, high fat, high calorie;

2 High vitamins and minerals, high fiber, low fat, low calorie.

**Table 2.** Multi-factorial logistic regression analyses for factors associated with students’ perceptions of their body image adjusted for all other factors in the Table.

**Factors**	**Body Image Perception**		
	**“Too Thin” OR (95% CI)^a^**	**“Just Right” OR (95% CI)^a^**	**“Too Fat” OR (95% CI)^a^**
**Gender**			
Females	1.00	1.00	1.00
Males	5.15 (3.10–8.57)	1.54 (1.16–2.04)	0.38 (0.29–0.50)
**Age (year)**			
<20	1.00	1.00	1.00
20–24	1.00 (0.53–1.88)	0.83 (0.56–1.21)	1.16 (0.80–1.69)
25–29	0.41 (0.14–1.22)	0.82 (0.49–1.37)	1.54 (0.93–2.55)
≥30	0.24 (0.80–0.68)	0.71 (0.47–1.08)	1.79 (1.19–2.69)
**University**			
Southern Denmark (SDU)	1.00	1.00	1.00
Chester (UC)	1.25 (0.70–2.25)	0.47 (0.34–0.66)	1.88 (1.36–2.61)
**Perceived stressors (high *vs.* low)**			
Studies in general	1.16 (0.89–1.52)	0.84 (0.65–1.10)	1.16 (0.89–1.52)
Workload in addition to studying	0.72 (0.39–1.31)	1.09 (0.80–1.48)	1.02 (0.75–1.38)
Housing	1.08 (0.48–2.47)	1.07 (0.68–1.66)	0.93 (0.61–1.44)
Financial situation	0.93 (0.56–1.52)	0.67 (0.50–0.88)	1.54 (1.17–2.04)
**Nutrition score**			
High calorie diet score^b^			
Low (1^st^ tertile)	1.00	1.00	1.00
Medium (2^nd^ tertile)	0.73 (0.44–1.24)	1.05 (0.76–1.44)	1.07 (0.79–1.47)
High (3^rd^ tertile)	0.35 (0.18–0.69)	1.29 (0.93–1.81)	1.04 (0.74–1.46)
Healthy diet score^c^			
Low (1^st^ tertile)	1.00	1.00	1.00
Medium (2^nd^ tertile)	0.59 (0.33–1.06)	1.48 (1.07–2.04)	0.83 (0.60–1.14)
High (3^rd^ tertile)	0.54 (0.30–0.99)	1.58 (1.15–2.16)	0.77 (0.56–1.05)
**Quality of life**			
Low	1.00	1.00	1.00
Medium	0.97 (0.36–2.64)	1.87 (1.04–3.35)	0.56 (0.32–0.99)
High	0.93 (0.36–2.41)	1.93 (1.09–3.42)	0.54 (0.31–0.93)
**Social support**			
High (≥3 persons)	1.00	1.00	1.00
Low (<3 persons)	1.04 (0.61–1.75)	1.08 (0.80–1.46)	0.92 (0.68–1.24)
**Satisfaction with social support**			
Low	1.00	1.00	1.00
Medium	1.06 (0.65–1.73)	0.89 (0.67–1.18)	1.12 (0.84–1.48)
High	1.50 (0.57–3.98)	1.32 (0.76–2.29)	0.69 (0.40–1.20)

a OR: odds ratio adjusted for all other factors in the table; CI: confidence interval;

b low vitamins and minerals, high fat, high calories, high carbohydrate;

c high vitamins and minerals, high fibre, low fat, low calories.
